# Automatic measurement of aortic annulus diameter in 3-dimensional Transoesophageal echocardiography

**DOI:** 10.1186/1471-2342-14-31

**Published:** 2014-09-08

**Authors:** Jørn Bersvendsen, Jan O Beitnes, Stig Urheim, Svend Aakhus, Eigil Samset

**Affiliations:** 1 GE Vingmed Ultrasound AS, Horten, Norway; 2 Department of Informatics, University of Oslo, Oslo, Norway; 3 Center for Cardiological Innovation, Oslo, Norway; 4 Department of Cardiology, Oslo University Hospital, Oslo, Norway

**Keywords:** Segmentation, Subdivision surface, 3-Dimensional Echocardiography, Aortic valve, Transcatheter aortic valve implantation

## Abstract

**Background:**

Transcatheter aortic valve implantation involves percutaneously implanting a biomechanical aortic valve to treat severe aortic stenosis. In order to select a proper device, precise sizing of the aortic valve annulus must be completed.

**Methods:**

In this paper, we describe a fully automatic segmentation method to measure the aortic annulus diameter in patients with aortic calcification, operating on 3-dimensional transesophageal echocardiographic images. The method is based on state estimation of a subdivision surface representation of the left ventricular outflow tract and aortic root. The state estimation is solved by an extended Kalman filter driven by edge detections normal to the subdivision surface.

**Results:**

The method was validated on echocardiographic recordings of 16 patients. Comparison against two manual measurements showed agreements (mean ±SD) of -0.3±1.6 and -0.2±2.3 mm for perimeter-derived diameters, compared to an interobserver agreement of -0.1±2.1 mm.

**Conclusions:**

With this study, we demonstrated the feasibility of an efficient and fully automatic measurement of the aortic annulus in patients with aortic disease. The algorithm robustly measured the aortic annulus diameter, providing measurements indistinguishable from those done by cardiologists.

## Background

Transcatheter Aortic Valve Implantation (TAVI) involves percutaneously implanting a biomechanical aortic valve to treat severe aortic stenosis. Because of its minimally invasive nature, TAVI is a viable alternative for patients who are at too high risk to undergo conventional surgical aortic valve replacement.

Precise sizing of the aortic annulus prior to TAVI is required for determining procedure eligibility and for selecting the correct implant size and type. Errors in prosthesis sizing may lead to complications during or after the procedure, such as Paravalvular Aortic Regurgitation (PAR) [1].

In current clinical practice, measurement of the annulus diameter before TAVI is typically done by 2D transthoracic echocardiography, 2D Transoesophageal Echocardiography (TEE) or Multi-Slice Computed Tomography (MSCT).

It has been shown that sizing based on MSCT, as opposed to 2D TEE, results in fewer instances of post operational PAR [2], as 2D modalities can fail to accurately describe the 3D structure of the aortic valve [3-5]. Strong correlations between 3D TEE and MSCT measurements of the annulus diameter [4] indicate feasibility of similar results for a method based on 3D TEE.

We propose an algorithm for automatic annulus measurements operating on 3D TEE images, using a real-time volumetric tracking and segmentation framework presented by Orderud et al. [6,7]. The framework uses an extended Kalman filter to solve a state space estimation formulation of the segmentation problem, and has been applied on the left ventricle.

In this paper, we apply the same framework to model the left ventricular outflow tract and aortic root. We propose a two-stage approach by performing segmentation based on a stiff and deformable surface sequentially. Combined with assimilation of forward and backward tracking, we obtain a fully automatic measurement of the aortic annulus diameter in 3D TEE images.

We validated our results by comparing automatic measurements of 16 recordings to manual measurements made by two cardiologists.

## Methods

### Segmentation

The method presented here is an application of a previously presented real-time volumetric segmentation framework, operating on deformable subdivision surfaces [6,7]. The segmentation is represented as a state estimation problem and solved with an extended Kalman filter.

**Figure 1:**
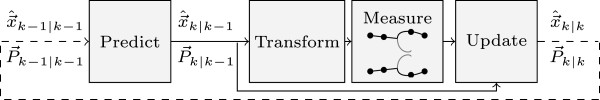
**State estimation KF processing chain.** Figure adapted from [6]. $\;{\hat{\text{x}}}_{k|j}$ and ***P***_*k*|*j*_ denote the state and covariance estimates respectively, at time index *k* using measurements up to and including time index *j*.

The filter is run iteratively over all frames in a heart cycle with a single iteration on each frame. For each frame, a motion model predicts the next estimate ${\hat{\text{x}}}_{k|k-1}$. Edge detection is then done locally on the deformed model surface, and the prediction is updated with the measurement information, resulting in the state estimate ${\hat{\text{x}}}_{k|k}$. This processing chain is illustrated in Figure 1.

#### *Surface model*

**Figure 2:**
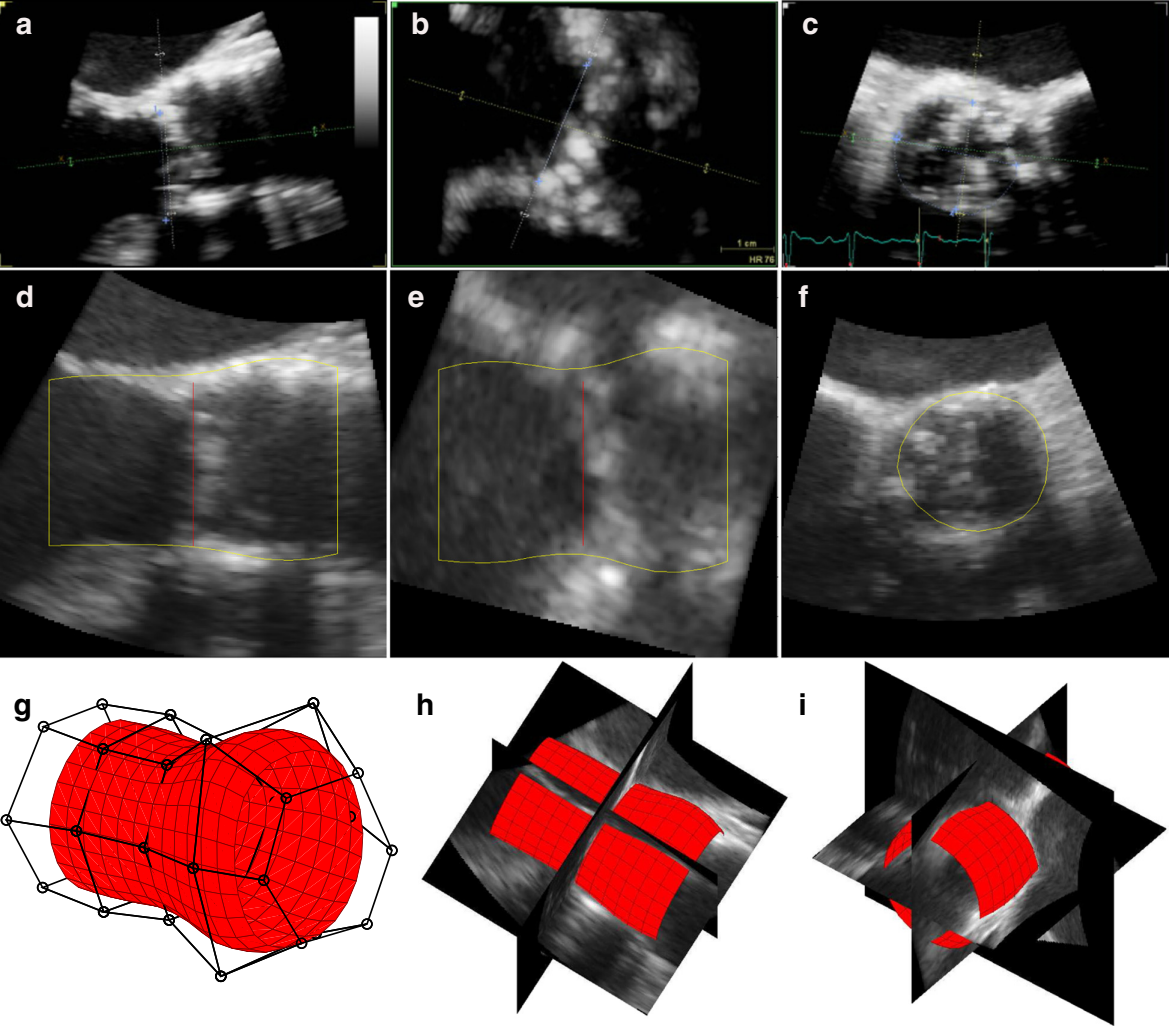
**Example measurements.** Manual **(a-c)** and automatic **(d-f)** measurements of the aortic valve annulus in a 3D TEE recording. Initial **(g)** and deformed **(h-i)** subdivision surface.

We use a cylindrical Doo-Sabin subdivision surface consisting of 5 connected circles of 6 uniformly distributed control points to represent the Left Ventricular Outflow Tract (LVOT) and aortic root, illustrated in Figure 2(g). A subdivision surface has the advantage of beging highly deformable but parameterized by only a few states, making the state space estimation an efficient approach.

The surface is deformed locally by displacing each control point in the direction normal to the cylinder long axis, to maximize deformation per degree of freedom. The local deformation transform is denoted **T**_*l*_(**p**;**x**_*l*_), where **x**_*l*_ is the state vector of local deformations.

A global transform

(1) $${\text{T}}_{g}(\text{p};{\text{x}}_{g})=s{\text{R}}_{x}(\theta_{x}){\text{R}}_{y}(\theta_{y}){\text{R}}_{z}(\theta_{z})\text{p}+{\left[\;t_{x},\;t_{y},\;t_{z}\right]}^{⊤}$$

where x_*g*_=[*t*_*x*_, *t*_*y*_, *t*_*z*_, *s*, *θ*_*x*_,*θ*_*y*_,*θ*_*z*_]^⊤^ is the global transform state vector, allows for translation, scaling and rotation of the model. The composite transform is given by **T**(**p**;**x**)=**T**_*g*_(**T**_*l*_(**p**;**x**_*l*_);**x**_*g*_) where ${\text{x}}^{⊤}=\left[{\text{x}}_{g}^{⊤},\;{\text{x}}_{l}^{⊤}\right]$ is the state vector.

The aortic annulus plane is represented by a disc placed in the middle of the surface model. The disc shares the same global transform **T**_*g*_ but is not deformable.

#### *Motion model*

The time domain dynamics of the model is inferred in the Kalman filter prediction step. We use a combination of the previous estimate ${\hat{\text{x}}}_{k-1|k-1}$ and a regularization state ${\hat{\text{x}}}_{0,k}$ to predict the next estimate by

(2) $${\hat{\text{x}}}_{k|k-1}=\text{A}{\hat{\text{x}}}_{k-1|k-1}+(\text{I}-\text{A}){\text{x}}_{0,k}\;,$$

where **A** is a diagonal matrix specifying the regularization strength for each state. Note that 0≤*a*_*ij*_≤1 ensures stability. The diagonal elements of **A** were chosen separately for translation, scaling, rotation and deformation states.

The covariance matrix of the estimate is predicted by

(3) $${\text{P}}_{k|k-1}={\text{AP}}_{k-1|k-1}{\text{A}}^{⊤}+{\text{Q}}_{0,k}\;,$$

where **Q**_0_ is the process noise covariance matrix. A low noise value will decrease the prediction covariance relative to the measurement covariance, which in turn will make the Kalman filter rely more on the previous estimate than the detected edges. **Q**_0_ therefore functions as a fairness parameter.

#### *Edge detection*

300 evenly distributed points on the model surface are defined. For the valve disc, 40 edge points are defined.

After applying **T**_*l*_, each edge point **p**_*l*_ is extracted with associated unit normal **n**_*l*_ and Jacobian matrix **J**_*l*_. These are then transformed to the global space by

(4) $${\text{p}}_{g}={\text{T}}_{g}({\text{p}}_{l};\hat{\text{x}})$$

(5) ng=|M|M-⊤nlwhereM=∂Tg(p;x)∂pplx^

(6) Jg=∂Tg(p;x)∂xplx^,∂Tg(p;x)∂pplx^Jl

where $\hat{\text{x}}={\hat{\text{x}}}_{k|k-1}$.

Edge displacements are detected by searching along **n**_*g*_ around **p**_*g*_ using the least mean squares fit to an intensity step or peak function. Outlier edges are rejected based on the intensity step function height and differences between neighboring edges. The capture range is determined by the search length along **n**_*g*_.

Each measured edge displacement *v*_*i*_ has an associated measurement noise with estimated variance *r*_*i*_ which is the sum of squared deviations in the intensity fit. The variance estimates are normalized such that $\underset{i}{\sum }r_{i}=r_{\text{edge}}$.

#### *Measurement update*

To relate the edge displacement *v*_*i*_ to changes in the state vector, the measurement vector ${\text{h}}_{i}^{⊤}={\text{n}}_{i}^{⊤}{\text{J}}_{i}$ is calculated, where **J**_*i*_ is the global Jacobian evaluated at **p**_*i*_ for ${\hat{\text{x}}}_{k|k-1}$. By assuming that all measurement noises are independent, the Kalman filter update step can be written as [6]

(7) $${\text{P}}_{k|k}^{-1}={\text{P}}_{k|k-1}^{-1}+\underset{i}{\sum }{\text{h}}_{i}r_{i}^{-1}{\text{h}}_{i}^{⊤}$$

(8) $${\hat{\text{x}}}_{k|k}={\hat{\text{x}}}_{k|k-1}+{\text{P}}_{k|k}\underset{i}{\sum }{\text{h}}_{i}r_{i}^{-1}v_{i}\;.$$

This computation is efficient as it does not require inversion of matrices with size dependent on the number of measurements.

#### *Forward and backward tracking*

A common problem with segmentation of time-series is that the segmentation lags behind the recording. We solve this by tracking forward and backward in time.

The Kalman filter is iterated forward over frames *k*=1,2,…,*N* to produce estimate x_*f*,*k*_ with estimated covariance **P**_*f*,*k*_. Backwards iteration over frames *k*=*N*,*N*-1,…,1 produces **x**_*b*,*k*_ and **P**_*b*,*k*_. The forward and backward state estimates are then assimilated by

(9) $${\text{P}}_{k}={\left({\text{P}}_{f,k}^{-1}+{\text{P}}_{b,k}^{-1}\right)}^{-1}$$

(10) $${\hat{\text{x}}}_{k}={\text{P}}_{k}\left({\text{P}}_{f,k}^{-1}{\hat{\text{x}}}_{f,k}+{\text{P}}_{b,k}^{-1}{\hat{\text{x}}}_{b,k}\right)\;.$$

This bidirectional tracking makes the segmentation robust to significant movement of the LVOT and aortic root during the cardiac cycle.

#### Two-phase segmentation

The described tracking algorithm is run in two passes; stiff segmentation and deformable segmentation.

##### Stiff segmentation

In the first pass, the subdivision surface is made stiff by removing all deformation states. The initial mesh is then oriented along an estimated LVOT long axis, derived from the ultrasound recorded roll angle.

The Kalman filter iterates over each consecutive frame once to ensure rough convergence. This is then repeated for the actual segmentation. A simplified motion model is used where **x**_0,*k*_ = **x**_0_ and **Q**_0,*k*_ = **Q**_0_ are constant during the cardiac cycle. **Q**_0_ was chosen to be a diagonal matrix of process noise standard deviations.

The resulting global pose states ${\hat{\text{x}}}_{\text{stiff}},k$, with estimated covariance **P**_stiff_,*k*, aligns the subdivision surface to the recording for each frame. This captures the global movement of the LVOT and aortic root during the cardiac cycle.

##### Deformable segmentation

After stiff segmentation, the deformation states are reintroduced. The state vectors ${\hat{x}}_{\text{stiff}},k$ and covariance matrices **P**_stiff_,*k* from the stiff segmentation are used for **x**_0,*k*_ and **Q**_0,*k*_ in the motion model. Bidirectional tracking is performed over a single heart cycle.

Different prediction parameters **A** ar e used in the stiff and deformable phases to reflect the increased confidence of the global transform states after stiff segmentation. For deformable segmentation, the regularization strength **A** is increased for the these states, ensuring that the movement of the aortic structure is tracked.

#### Automatic annulus measurements

The aortic annulus is extracted by the intersection of the deformed surface model and the aortic valve disk. An ellipse is fitted to the intersection points by least mean squares optimization and the major and minor axes, area and perimeter are extracted.

The mid systolic frame was defined as the frame with maximum detected aortic annulus area.

### Transoesophageal echocardiography

#### Acquisition

16 anonymous 3D TEE recordings were provided retrospectively by the Oslo University Hospital for validation. Patient characteristics are shown in Table 1. The dataset contained both tri- and bicuspid aortic valves with varying degrees of stenosis and insufficiency. The images were acquired in mid-esophageal position using zoom mode and showed the LVOT, aortic valve and aortic root. All images were recorded on a Vivid E9 scanner with a 6 VT-D probe and all analysis was done using EchoPAC version 112.1.0 (GE Vingmed Ultrasound AS, Horten Norway).

**Table 1:** Patient characteristics

	
Age ^∗^, yr	69±16
Sex ^∗^, male/female	7/6
Disease, n	
Aortic stenosis	12
Aortic insufficiency	2
Normal	2
Aortic morphology, n	
Tricuspid	14
Bicuspid	2
LV EF < 50 % ^∗^, n	3

^∗^Age, sex and LV EF were unknown for 3 patients.

#### *Manual measurements*

**Figure 3:**
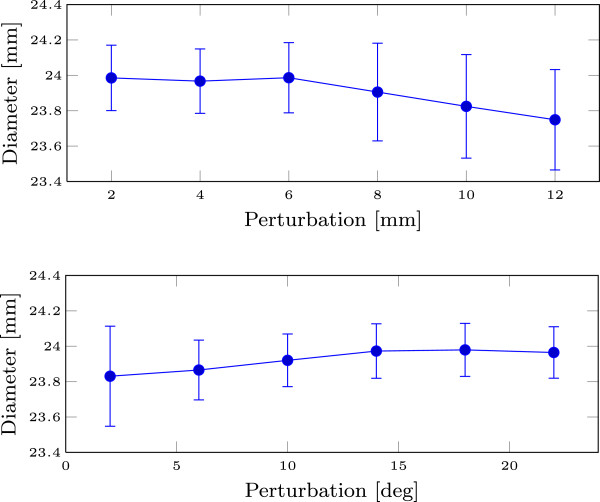
**Sensitivity analysis results.** The plot shows the mean and standard deviation of the output diameter distributions as a function of translational and rotational perturbations.

The aortic annulus was manually measured by two cardiologists. The annulus plane was visualized using 3 orthogonal planes in mid systole. A sagittal and a coronal plane bisected the long axis of the LVOT and a transverse plane bisected the lowest insertion points of all 3 aortic cusps, as shown in Figure 3(a-c). The annulus diameter was measured in the sagittal and coronal long axis planes. Area and perimeter were measured by manual trace in the short axis plane. The manual observers where blinded to each other and the results from the automatic measurements.

### Sensitivity analysis

To assess the robustness of the algorithm with respect to the roll-angle derived initial LVOT long axis estimate, the following test was carried out. For a single recording, the initial state **x**_0_ prior to stiff segmentation was randomly perturbed 500 times, and the resulting perimeter-derived annulus diameter distributions were recorded. This was done separately for perturbations to translation and rotation.

**Translation** The perturbations were on the form *d***n**/||**n**|| where the elements of $\text{n}\in R^{3}$ were uniformely distributed. The analysis was repeated for each *d*=2,4,…,12 mm, which is within the capture range of ± 14 mm.**Rotation** For each iteration, a vector **n**/||**n**|| was generated, where the elements of $\text{n}\in R^{3}$ were uniformely distributed. The initial model was then rotated an angle *ϕ* about the line **v**(*t*)=**v**_0_+**n***t* where **v**_0_ is the initial annulus center. The analysis was repeated for each *ϕ*=1,2,…,15 deg.

To assess robustness with respect to scaling, the segmentation was repeated for 100 linearly spaced initial annulus diameters *D*_0_∈[ 15,35] mm.

### Statistical analysis

Agreement between the automatic method and the two manual observers was analyzed using Blant and Altmans method and two-way absolute agreement intraclass correlation coefficients. All statistical analysis was performed using IBM SPSS Statistics for Windows, Version 20.0 (IBM Corp., Armonk, NY, USA).

## Results

The described algorithm was executed on all 16 3D TEE datasets. The segmentation time was (mean ±SD) 9.9±7.3 s on a standard laptop.

### Sensitivity analysis

Figure 3 shows the results of the sensitivity analysis for translational and rotational perturbations in the initial state. The sample standard deviations of the resulting diameter distributions were ≤0.29, ≤0.28 and 0.10 mm for the translation, rotation and scaling sensitivity tests respectively.

### Comparison of manual and automatic measurements

**Figure 4:**
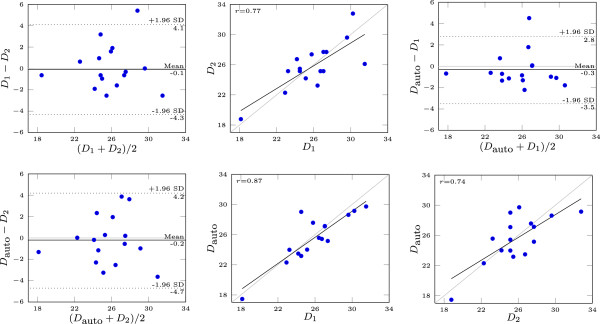
**Comparison of automatic and manual perimeter-derived measurements.** All diameters are [mm]. *D*_*auto*_, *D*_1_ and *D*_2_ denote automatic and manual measurements from the the first and second observer respectively.

Comparisons of manual and automatic measurements are shown in Tables 2, 3 and Figure 4. Figure 2 shows an example of the manual and automatic measurements.

**Table 2:** Automatic and manual measurements of arotic annulus diameters

	***D***_**auto**_	***D***_**1**_	***D***_**2**_
Minimum	24.3 ± 3.0		
Maximum	26.8 ± 3.5		
Sagittal		23.9 ± 2.7	23.9 ± 3.1
Coronal		25.4 ± 3.3	25.3 ± 3.0
Area-derived	25.5 ± 3.2	24.9 ± 3.0	25.0 ± 3.1
Perimeter-derived	25.6 ± 3.2	25.9 ± 3.2	25.8 ± 3.1

Values are mean ±SD [mm]. *D*_auto_, *D*_1_ and *D*_2_ denote automatic and manual measurements from the the first and second observer respectively.

**Table 3:** Comparison of automatic and manual measurements of arotic annulus diameter

	**Bias**	**Intraclass correlation**
Sagittal		
*D*_1_ versus *D*_2_	0.063 ± 1.5	0.88
Coronal		
*D*_1_ versus *D*_2_	0.063 ± 2.4	0.74
Perimeter-derived		
*D*_*auto*_ versus *D*_1_	-0.35 ± 1.6	0.87
*D*_*auto*_ versus *D*_2_	-0.23 ± 2.3	0.75
*D*_1_ versus *D*_2_	0.12 ± 2.1	0.78
Area-derived		
*D*_*auto*_ versus *D*_1_	0.62 ± 1.7	0.85
*D*_*auto*_ versus *D*_2_	0.46 ± 2.3	0.74
*D*_1_ versus *D*_2_	-0.16 ± 2.1	0.77

Biases are mean ±SD [mm]. *D*_auto_, *D*_1_ and *D*_2_ denote automatic and manual measurements from the the first and second observer respectively.

The interobserver Intraclass Correlation Coefficients (ICC) were 0.78 and 0.77 for perimeter and area derived diameters respectively. The intraclass correlation coefficients between the automatic method and each of the manual observers were 0.87 and 0.75 for the perimeter derived diameters and 0.85 and 0.74 for the area derived diameters.

## Discussion

### Sensitivity analysis

Even for significant perturbations (± 12 mm translation, ± 15 deg rotation or ± 10 mm initial annulus diameter), the standard deviation of the automatic measurement was significantly lower than the interobserver variation. This indicates that the algorithm is robust with respect to errors in the initial LVOT long axis derived from the recorded roll angle, as well as the assumed initial annulus diameter.

### Comparison of manual and automatic measurements

The algorithm performance was indistinguishable from human observers’ performance. The automatic algorithm successfully segmented the LVOT and aortic root and measured the aortic annulus diameter in all 16 images, with mean computation time 9.9 s. Interobserver correlation coefficient for the manual measurements was comparable to that reported by others [4]. The correlation and deviation between the automatic and each manual measurements were comparable to the interobserver reliability.

Perimeter-derived measurements showed the closest agreement with the manual observers. Since the perimeter-derived diameter changes the least during the cardiac cycle [8], this measurement should not be affected by errors in mid-systole detection. This is therefore a more robust measurement compared to area, major and minor diameters, and was chosen as the algorithm’s main output diameter.

The annulus plane is normally defined as the plane spanned by the hinge points of the three valve cusps. However, the hinge points are not explicitly defined in the described model. Because the disk representing the aortic valve is non-deformable and shares the same pose transform as the surface model, the detected annulus will align perpendicular to the long axis of the LVOT.

Since the hinge point plane and the perpendicular plane are closely aligned, we propose that a perpendicular plane is a good estimation of the anatomical annulus plane. In the rare cases where these planes are not aligned, we submit that a perpendicular plane is of clinical relevance since a prosthetic valve is more likely to align with the LVOT long axis than the native valve’s hinge points.

The largest absolute deviation between the two manual observers was 5.4 mm. Poor image quality, low frame rate (7.7 vps) and a wide sinus of Valsalva lead to the significant interobserver deviation. The largest absolute deviation between automatic and manual measurements was 5.5 mm. In this case, the automatic method grossly overestimated the annulus diameter, resulting from a very wide sinus of Valsalva combined with dropouts close to the annulus. Although these discrepancies would indicate a difference in device selection, it is extremely unlikely that these specific images would be used as the basis of device selection.

Two recordings had visible stitching artifacts. In both recordings all absolute deviations between the the automatic and manual measurements were ≤0.6 mm, indicating robustness against stitching artifacts.

Recently, a validation study of the first description of an automated aortic root modeling and quantification algorithm for 3D TEE images was published [9]. The study reported annulus diameter agreement (mean ±SD) of 1.1±1.3 and 3.6±2.3 mm for sagittal and coronal diameters respectively, which is comparable to our results. However, manual identification of peak systole and end diastole was required, and manual segmentation adjustments were needed in 23 of 69 TEE recordings. The reported interobserver variability was 0.2±0.56 and 0.0±0.61 mm. Although our presented algorithm is fully automatic and therefore has no interobserver variability, these values are comparable to our sensitivity analysis results. The reported computation and adjustment time was 2.3±0.6 minutes, which is significantly longer than our results.

This method is based on machine learning and statistical shape models [10]. However, these algorithms require a large database of recordings annotated with manual ground truth segmentations. The presented method is simpler and does not rely on a history of previous segmentations.

Within the presented framework, there are several quality measures available that can potentially be used to automatically identify poor segmentations, e.g. number of discarded edge profiles, deviation from segmentation surface to detected edges or the state covariance estimates. These should be further investigated with an available gold standard to create criteria that can automatically judge the segmentation quality.

This study used a limited sample size of 16 patients. Further studies with a larger number of patients should be performed.

Comparison of measurements in prospective 3D TEE images with ECG gated Multi-Slice Computed Tomography (MSCT) gold standard should be carried out to investigate if the algorithm can render MSCT superfluous for a significant portion of TAVI candidates.

## Conclusions

With this study, we demonstrated the feasibility of an efficient and fully automatic measurement of the aortic annulus in patients with aortic disease. The algorithm robustly measured the aortic annulus diameter, providing measurements indistinguishable from those done by cardiologists.

## Competing interests

JB and ES are employed in GE Vingmed Ultrasound AS.

## Authors’ contributions

JB designed and implemented the algorithm, performed the statistical analysis, participated in the study design and drafted the paper. JOB and SU performed the manual measurements, participated in the study design and in drafting the manuscript. SA conceived of the study, participated in its design and in drafting the manuscript. ES conceived of the study, participated in the algorithm design, study design and in drafting the manuscript. All authors read and approved the final manuscript.

## Pre-publication history

The pre-publication history for this paper can be accessed here:

http://www.biomedcentral.com/1471-2342/14/31/prepub
